# Omics-Based Identification of Shared and Gender Disparity Routes in *Hras12V*-Induced Hepatocarcinogenesis: An Important Role for Dlk1-Dio3 Genomic Imprinting Region

**DOI:** 10.3389/fgene.2021.620594

**Published:** 2021-05-31

**Authors:** Jing Zhang, Huiling Li, Jianyi Dong, Nan Zhang, Yang Liu, Xiaoqin Luo, Jun Chen, Jingyu Wang, Aiguo Wang

**Affiliations:** Department of Comparative Medicine, Laboratory Animal Center, Dalian Medical University, Dalian, China

**Keywords:** miRNAomics, hepatocarcinogenesis, gender disparity, Dlk1-Dio3 genomic imprinting region, *Hras12V*-transgenic mice

## Abstract

The phenomenon of gender disparity is very profound in hepatocellular carcinoma (HCC). Although previous research has revealed important roles of microRNA (miRNA) in HCC, there are no studies investigating the role of miRNAs in gender disparity observed hepatocarcinogenesis. In the present study, we investigated the global miRNAomics changes related to *Ras*-induced male-prevalent hepatocarcinogenesis in a *Hras12V*-transgenic mouse model (*Ras*-Tg) by next-generation sequencing (NGS). We identified shared by also unique changes in miRNA expression profiles in gender-dependent hepatocarcinogenesis. Two hundred sixty-four differentially expressed miRNAs (DEMIRs) with *q* value ≤0.05 and fold change ≥2 were identified. A vertical comparison revealed that the lower numbers of DEMIRs in the hepatic tumor (T) compared with the peri-tumor precancerous tissue (P) of *Ras*-Tg and normal liver tissue of wild-type C57BL/6J mice (W) in males indicated that males are more susceptible to develop HCC. The expression pattern analysis revealed 43 common HCC-related miRNAs and 4 *Ras*-positive-related miRNAs between males and females. By integrating the mRNA transcriptomic data and using 3-node FFL analysis, a group of significant components commonly contributing to HCC between sexes were filtered out. A horizontal comparison showed that the majority of DEMIRs are located in the Dlk1-Dio3 genomic imprinting region (GIR) and that they are closely related to not only hepatic tumorigenesis but also to gender disparity in hepatocarcinogenesis. This is achieved by regulating multiple metabolic pathways, including retinol, bile acid, and steroid hormones. In conclusion, the identification of shared and gender-dependent DEMIRs in hepatocarcinogenesis provides valuable insights into the mechanisms that contribute to male-biased *Ras*-induced hepatic carcinogenesis.

## Introduction

Hepatocellular carcinoma (HCC) is an extremely heterogeneous cancer; treatment options are limited, and its prognosis remains extremely poor. A male predominance in HCC incidence has been reported, with male-to-female ratios ranging between 2:1 and 8:1 (Wang et al., 2006; Hartwell et al., 2014; El-Serag, 2012). Consistently, animal studies also revealed that hepatocarcinogenesis is more common in male rodents, regardless of its etiology (spontaneous, or induced chemically, genetically, or through chronic viral infection) (Nakatani et al., 2001; Naugler et al., 2007; Brandon-Warner et al., 2012). Therefore, humans and animals may share common mechanisms contributing to the gender disparity of hepatocarcinogenesis; these mechanisms remain, however, largely unknown.

MicroRNAs (MiRNAs) are small non-coding RNA molecules, consisting of approximately 22 nucleotides. They are conserved among several organisms, including plants, animals, and viruses. It is now clear that miRNAs they are involved in post-transcriptional regulation of gene expression (Bartel, 2004). It is becoming increasingly evident that miRNAs are involved in cancer development and progression, by exerting anti-tumor or tumor-promoting roles (Ji et al., 2017). The abnormal expression of miRNAs is involved in several biological processes, including proliferation, apoptosis, and metastasis, and could serve as therapeutic targets and prognostic biomarkers for HCC (Maluccio and Covey, 2012). However, there are no studies on the gender disparity of miRNA expression profiles related to hepatocarcinogenesis.

*RAS* gene is commonly mutated in the vast majority of all human tumors; their frequency is the highest among all genes associated with human cancers (Hunter, 1997). Moreover, *Ras* is mutated in 70% of murine HCCs. Despite the fact that activating mutations in *RAS* occur in only 5% of human HCC, the aberrant activation of the RAS/MAPK signal transduction pathway due to other mutations is very common in human HCC (Newell et al., 2009; Taketomi et al., 2013; Delire and Starkel, 2015), supporting the notion that RAS plays a key role in hepatocarcinogenesis (Wang et al., 2005). We have previously generated a *Hras12V* transgenic mouse line using an *Hras12V*-encoding construct, which provides a hepatocyte-specific expression of the *Ras* oncogene, resulting in multicentric spontaneous hepatic tumorigenesis which is highly reproducible and shows male prevalence. The hepatic tumorigenesis in *Hras12V* transgenic mouse is primarily initiated by Ras/MAPK pathway. However, multiple pathways are involved in hepatic tumor development, such as PI3K/AKT/mROR, PPAR signaling pathway, et al. (Wang et al., 2005; Li et al., 2020). This hepatic tumor animal model has led to the elucidation of several mechanisms underlying hepatocarcinogenesis (Fan et al., 2017; Rong et al., 2017).

In the present study, we employed the *Hras12V* transgenic mice and next-generation sequencing (NGS) to investigate the global miRNAomics profile related to *Ras*-induced hepatic tumorigenesis with gender disparity. Moreover, by vertical comparison within males and females and horizontal comparisons between sexes, we identified shared, unique, and systemic signatures in hepatocarcinogenesis. Especially, the importance of Dlk1-Dio3 genomic imprinting region (GIR) in hepatocarcinogenesis was found. In addition, by integrating transcriptomics data and using 3-node FFL analysis, a group of significant components contributing to HCC among sexes were identified.

## Results

### Next-Generation Sequencing Identified Gender-Dependent miRNA Expression Profiles in Hepatocarcinogenesis

**FIGURE 1:**
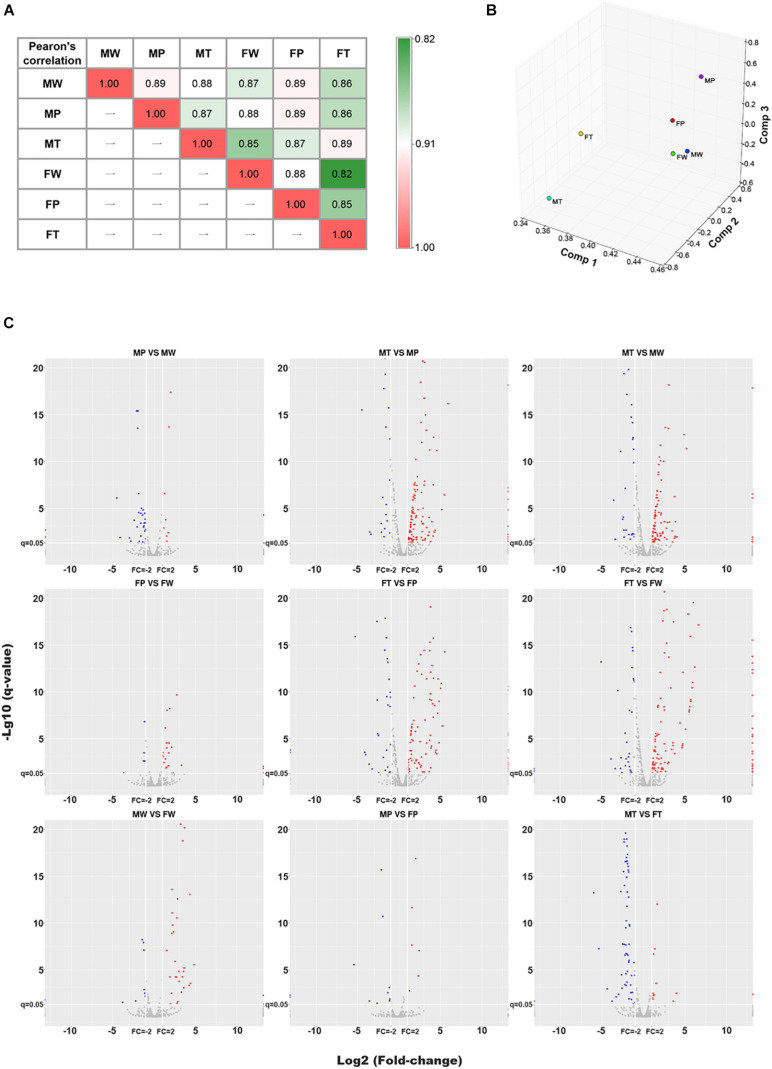
The Pearson’s correlation, PCA and DEMIRs analysis for microRNA omics data. **(A)** Heatmap of the Pearson’s correlation (R2) analysis of miRNAomics dataset. Values denote the Pearson correlation coefficients. **(B)** Principal component analysis (PCA) calculation results are expressed as 3D scatter plot. **(C)** Volcano plot of pairwise comparisons. Red and blue colors represent relatively up- and down-regulated miRNAs, respectively. MW and FW, wild-type liver tissues of males and females, respectively; MP and FP, peri-tumor tissues of males and females, respectively; MT and FT, hepatic tumor tissues of males and females, respectively.

To detect miRNA expression profiling, the normal liver tissue samples (W) of wild-type non-transgenic mice (non-Tg) and hepatic tumors (T) and matched adjacent precancerous tissue samples (P) of *Hras12V* transgenic mice (*Ras*-Tg) from males and females were collected and named MW, MP, MT, FW, FP, and FT (M indicates male; F indicates female), respectively (Supplementary Figure 1). The miRNA expression profiles of these samples were assessed by NGS technology from isolated total RNAs. For every sample, an average of 27 million (ranged from 20 to 36 million) miRNA raw reads were obtained and the average clean ratio for the raw reads was 98.87% (ranged from 97.97 to 99.48%) (Supplementary Table 1). Saturation analysis showed that the miRNAs sequenced and mapped by trimmed reads were saturated when the sequencing depth approached 10 million (Supplementary Figure 2), indicating that the sequencing depth used in our study was sufficient to achieved high transcriptome coverage. Pearson’s correlation and principal component analysis (PCA) indicated that the miRNA expression profiles of the six tissue groups vary significantly (Figures 1A,B). The miRNAs read values were transformed to TPM (transcripts per million), and, by using the criteria of *q*-value≤ 0.05 and fold-change ≥ 2, a total of 191 DEMIRs in males and 204 DEMIRs in females (Figure 1C and Supplementary Table 2) were identified at least in one paired comparison among W, P, and T. The identified miRNA expression profiling data revealed shared, but also unique expression patterns in hepatocarcinogenesis between sexes.

**FIGURE 2:**
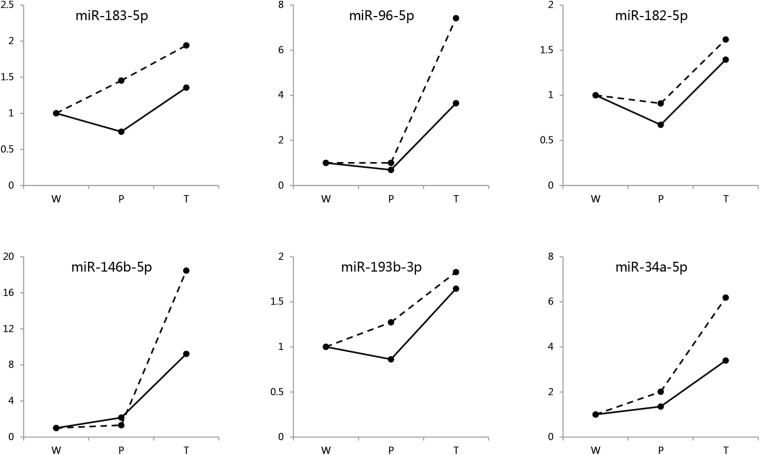
Validation for DEMIRs by RT-qPCR. Six randomly selected DEMIRs detected in both sexes were evaluated by RT-qPCR analysis. The primer sequences were presented in Supplementary Table 2.1. Hidden line, males; Solid line, females.

To assess the reliability of the quantitative miRNAomics analysis obtained by NGS, six of the identified differentially expressed miRNAs in both sexes were randomly selected and further evaluated in different males and females by quantitative real-time PCR (RT-qPCR; Figure 2). The expression changes of these miRNAs were consistent with the NGS data, both in males and in females (Supplementary Table 2).

### MiRNA Expression Profiling Revealed Different Distribution of DEMIRs During Hepatocarcinogenesis Between Sexes

**FIGURE 3:**
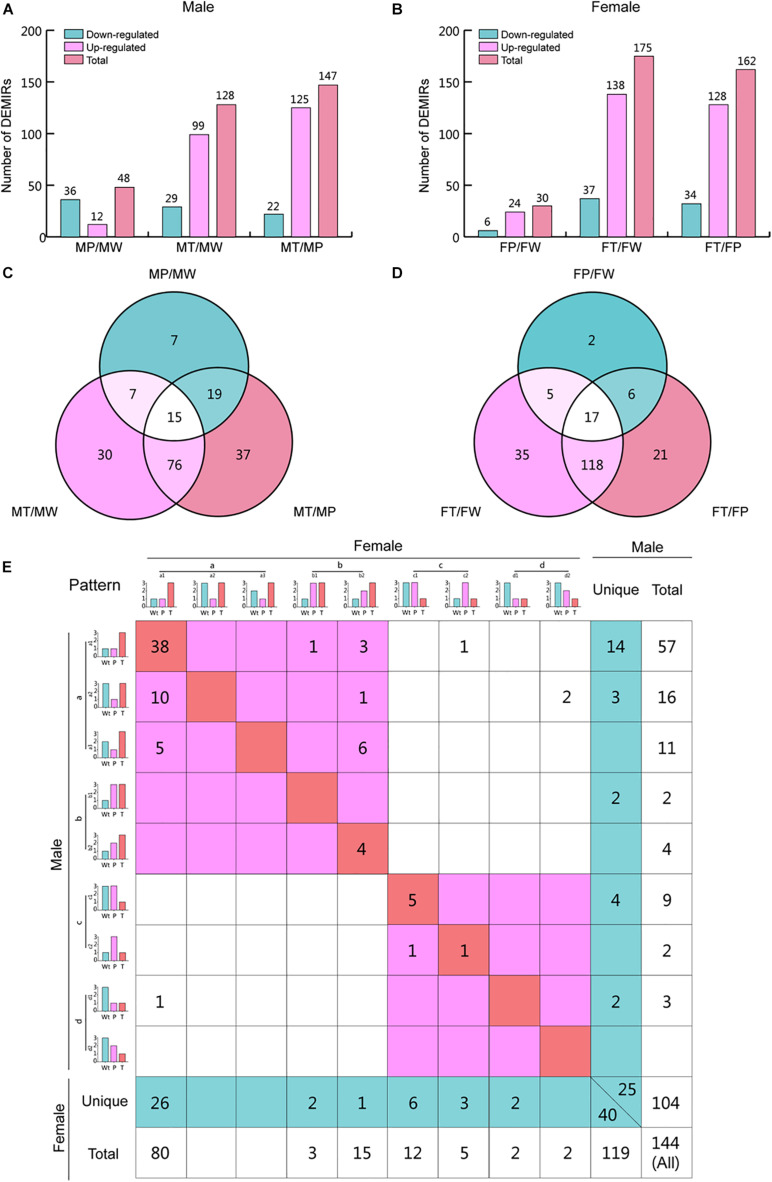
Differentially expressed miRNAs (DEMIRs) obtained by vertical comparison and expression pattern analysis for hepatocarcinogenesis. **(A)** The number of miRNAs that are expressed at higher or lower levels when comparing MP/MW, MT/MW, and MT/MP in male mice. **(B)** The number of up- and down-regulated miRNAs when comparing FP/FW, FT/FW, and FT/FP in female mice. **(C)** Venn analysis of the DEMIRs shown in Supplementary Table 2.1. **(D)** Venn analysis of the DEMIRs shown in Supplementary Table 2.2. **(E)** Expression pattern analysis for hepatocarcinogenesis for both sexes. The diagonal line indicates that these miRNAs have the same expression patterns in both sexes. The miRNAs shown in the red area in the upper left are up-regulated in T as compared to P and/or W in both sexes. The miRNAs shown in the red area in the bottom right are down-regulated in T as compared to P and/or W in both sexes. The miRNAs shown in the blue area have a unique expression pattern in males and females. The relative expression levels of miRNAs are represented using a 1, 2, and 3 grading system, indicating significant differences in the miRNA levels. The DEMIRs that appeared at least two times in pairwise comparison among W, P, and T between sexes are shown here, and the expression pattern analysis for total DEMIRs is shown in Supplementary Figure 3.

To investigate the changes in miRNA expression profiles during hepatocarcinogenesis in each sex, we determined the numbers of miRNAs that their expression changed significantly, by pairwise comparison among W, P, and T of males and females. Interestingly, the changes in miRNA expression profiles differed between males and females (Figures 3A,B). For both sexes, the most prominent changes were observed when T was compared with W and P, indicating that miRNAs may play crucial roles in hepatocarcinogenesis. In T versus W (T/W) and T versus P (T/P), the numbers of up-regulated miRNAs were approximately 3 to 4-fold higher than that of down-regulated miRNAs, implying that HCC-related miRNAs play a main role in the down-regulation of protein levels. Profoundly higher numbers of miRNAs whose expression was altered in T was observed in females, indicating that the aberrant regulation of a higher number of miRNAs is needed for hepatic transformation in females, reflecting the lower incidence of HCC observed in females. On the other hand, the number of miRNAs whose expression was altered in P versus W (P/W) was higher in males, indicating that males are more susceptible to *Ras*-induced carcinogenesis. The opposite changes in numbers of up- and down-regulated miRNAs in P/W suggests that different mechanisms are involved in oncogene stress in males and females.

### Shared and Unique miRNAs Expression Patterns During Hepatocarcinogenesis Between Sexes

To identify shared and unique miRNAs involved in hepatic tumorigenesis in males and females, Venn analysis was firstly performed for T/P, T/W, and P/W in males and females (Figures 3C,D and Supplementary Table 2). And then, we classified the miRNAs into four categories [from (a) to (b)] describing particular variation trends of miRNAs during hepatic tumorigenesis. Further, shared and unique miRNAs were identified, depending on the categories (Supplementary Table 3). To focus on the clear and definite variation tendency, we summarized at least two times significantly changed miRNAs in pairwise comparison among W, P, and T (Figure 3E) (Supplementary Figure 3 summarized the expression patterns for all detected 264 DEMIRs).

In total, 144 miRNAs were found to show at least two times significant changes in a pairwise comparison between W, P, and T (Figure 3E). Among them, 104 miRNAs were found in males, while 119 were found in females. There were 79 miRNAs shared to both sexes, while 25 and 40 miRNAs were unique in males and females, respectively. Among the shared miRNAs, 68 miRNAs were negatively associated with liver tumorigenesis. Among the unique miRNAs, 19 and 29 miRNAs were positively correlated to liver carcinogenesis in males and females, respectively, and 6 and 11 miRNAs were negatively correlated to hepatic tumors in males and females, respectively (Figure 3E and Supplementary Table 3).

Additionally, the four categories of miRNA expression patterns can be further classified into several subtypes (Figure 3E). The symbols used here indicate that “>” and “<”: miRNAs were significantly up- and down-regulated, respectively; and “=”: no significant difference in miRNA expression levels was observed between the samples. The category HCC-positive-related miRNAs (a) includes three subtypes: (1) T > P = W; (2) T = W > P; (3) T > W > P. The most miRNAs in this category were classified into subtype (1). The category *Ras*-positive-related miRNAs (b) includes two subtypes: (1) W < P = T and (2) W < P < T. These miRNAs were equally (or gradually) and significantly up-regulated in P and T compared with W. The category HCC-negative-related miRNAs (c) includes two subtypes: (1) T < P = W, (2) T = W < P. The most miRNAs in this category were classified into subtype (1). The category *Ras*-negative-related miRNAs (d) includes two types: (1) W > T = P and (2) W > P > T. These miRNAs were equally (or gradually) and significantly down-regulated in P and T compared with W.

In particular, 38 miRNAs in (a)-(1) type and 5 miRNAs in (c)-(1) type occupy the most proportions of miRNAs in variant trend types, which represent the shared miRNAs involved in hepatic tumor development (Supplementary Table 3). Additionally, 4 (in b) common miRNAs in both sexes were found to be positively related to *Ras* oncogene expression. Moreover, the higher number of unique miRNAs in females compared to males (40 versus 25) suggests that more changes in miRNAs expression are required in females to develop HCC (Supplementary Table 3).

### Identification of the Regulatory Networks That Are Involved in Hepatocarcinogenesis Both in Males and Females

**FIGURE 4:**
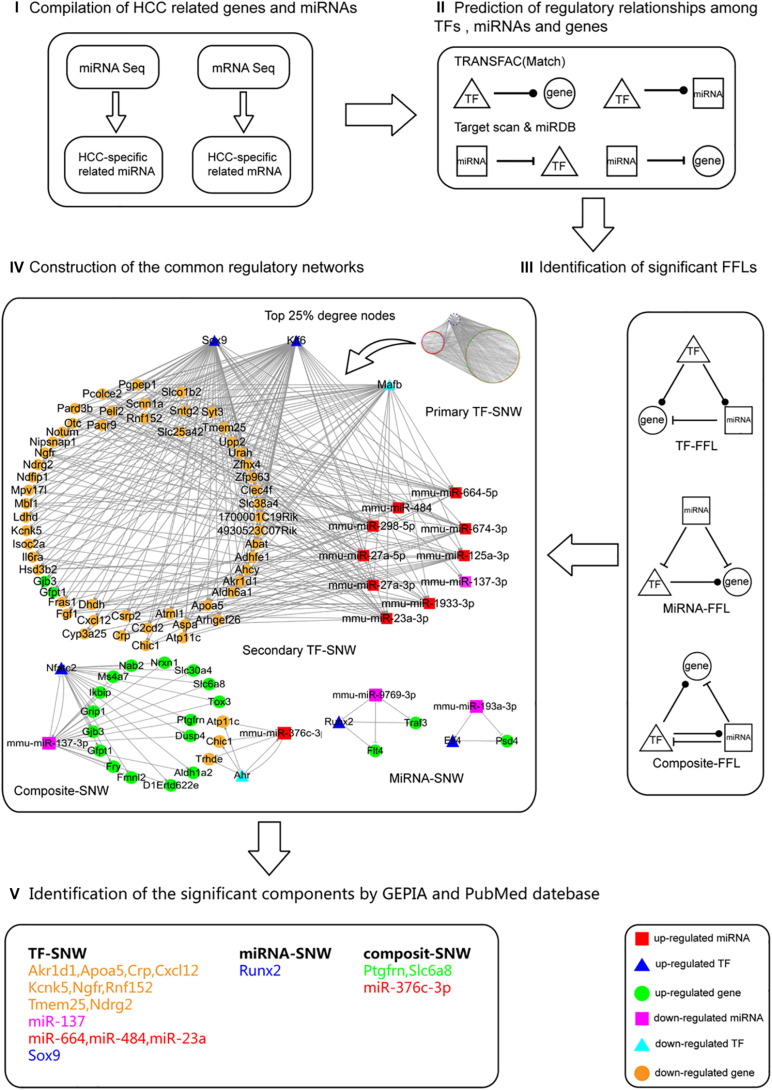
The process followed to construct the common regulatory networks and to identify the significant components involved in hepatocarcinogenesis. The process followed contained five steps. **(I)** Data compilation. The HCC commonly related miRNAs [38 up-regulated (T > P = W); 5 down-regulated (T < P = W)] and mRNAs [646 up-regulated (T > P = W); 323 down-regulated (T < P = W)] in both males and females were selected for further analysis. **(II)** Prediction of the regulatory relationships between TFs, miRNAs, and genes. The four regulatory relationships include TF-gene, TF-miRNA, miRNA-gene, miRNA-TF. **(III)** Identification of significant FFLs. Based on the above regulatory relationships, the significant 3-node FFLs were identified. **(IV)** Construction of the shared regulatory networks. These FFLs were categorized into TF, miRNA, and composite sub-networks (SNWs). Among these three SNWs, TF-SNW is the most redundant. By using the hub definition method, we determined the top 25% degree nodes of genes, miRNAs, and TFs as the member of secondary TF-SNW to focus on the most significant components. **(V)** Identification of the significant components using GEPIA and PubMed database. All nodes in the three SNWs (secondary TF-SNW, miRNA-SNW, composite-SNW) were filtered based on the GEPIA and PubMed database.

To identify the regulatory networks that are involved in hepatocarcinogenesis both in males and females, the common HCC-related miRNAs (38 up-regulated (T > P = W); 5 down-regulated (T < P = W) in Figure 3E) and mRNAs [646 up-regulated (T > P = W); 323 down-regulated (T < P = W) in Supplementary Table 4] in males and females were selected for further analysis (Figure 4I). Considering that mRNAs are negatively regulated by miRNAs, 323 down-expressed mRNAs may be regulated by 38 up-expressed miRNAs and 646 up-expressed mRNAs may results from 5 down-expressed miRNAs. Further, 35 TFs (27 in up-expressed and 8 in down-expressed mRNAs) were identified. Finally, four types of regulatory relationships among miRNAs, TFs, and genes were predicted (Figure 4II and Supplementary Tables 5, 6).

To merge the four types of regulatory relationships predicted above (Figure 4II), 3-node FFLs were formed (Figure 4III and Supplementary Tables 7, 8). The network contained 1,182 edges and 326 unique nodes in the 920 FFLs. Among the 1,182 edges, 461 miRNA-gene, 4 miRNA-TF, 588 TF-gene, and 129 TF-miRNA pairs were predicted. Among the 326 nodes, 265 genes, 46 miRNAs, and 15 TFs were specific for HCC. Further, these FFLs were categorized into TF-FFLs (TF), miRNA-FFLs (miRNA), and composite-FFLs (composite) sub-networks (SNW) (Figure 4IV). In these three SNWs, TF-SNW is the most redundant. By using the hub definition method proposed by Yu et al. (2004), we determined the top 25% degree nodes of gene, miRNA and TF as the member of secondary TF-SNW to focus on the most significant components. To further analyze the potential function, all nodes in the three SNWs (secondary TF-SNW, miRNA-SNW, composite-SNW) were filtered based on the GEPIA and PubMed database, identifying a group of significant components (Figure 4V).

### Differentially Expressed miRNAs Between Males and Females

**FIGURE 5:**
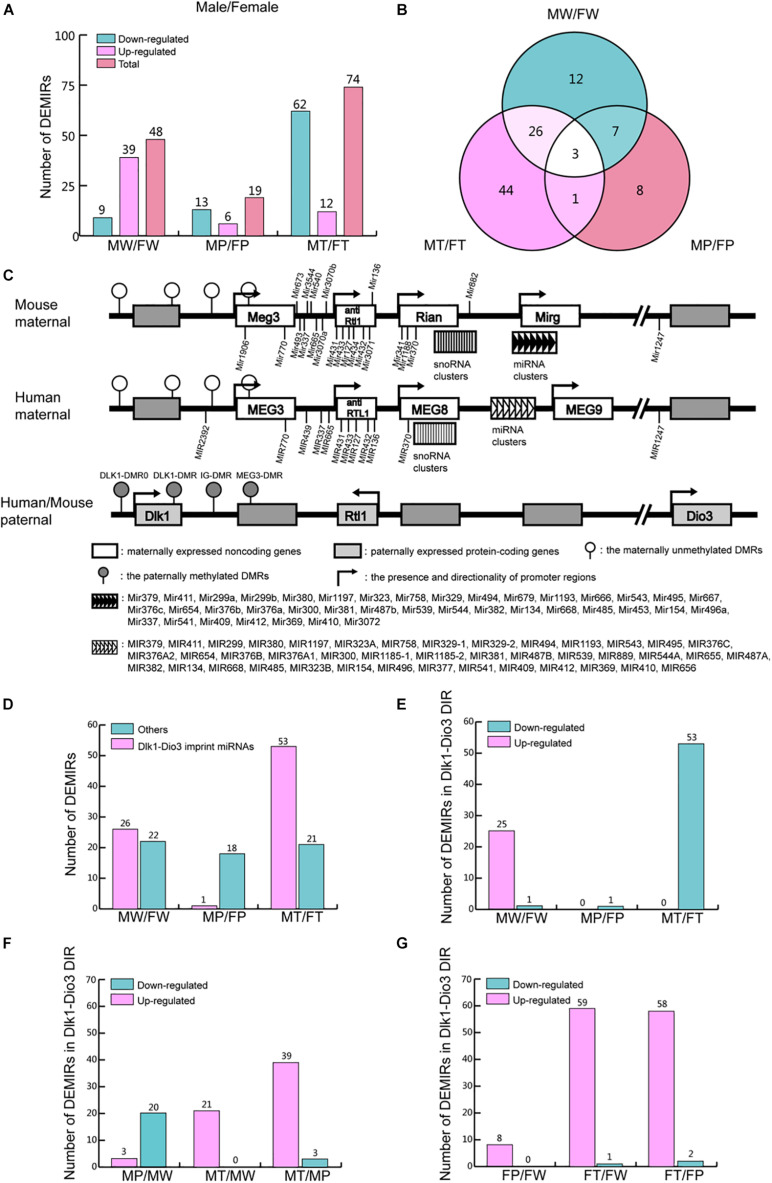
DEMIRs between sexes in the process of hepatocarcinogenesis. **(A)** The number of up- and down-regulated miRNAs in the comparisons in MW/FW, MP/FP, and MT/FT between sexes. **(B)** Venn analysis of DEMIRs in panel **(A)**. **(C)** Comparative structure model of Dlk1-Dio3 genomic imprinting region (GIR) between human and mice. **(D)** The number of DEMIRs located in and beyond Dlk1-Dio3 GIR. **(E)** The number of up- and down-regulated miRNAs located on Dlk1-Dio3 GIR between sexes. **(F)** The number of up- and down-regulated miRNAs located on Dlk1-Dio3 GIR in males. **(G)** The number of up- and down-regulated miRNAs located on Dlk1-Dio3 GIR in females.

To identify the DEMIRs between males and females involved in hepatic tumorigenesis, comparisons between the sexes [MW versus FW (MW/FW); MP versus FP (MP/FP); MT versus FT (MT/FT)] were performed. Interestingly, the highest number of DEMIRs was detected in MT/FT, and the lowest number of DEMIRs was detected in MP/FP (74 versus 19), whereas 48 DEMIRs were identified in MW/FW (Figure 5A). Venn diagram showed that the number of overlapped DEMIRs between MT/FT and MW/FW was higher than that between MP/FP and MT/FT (MW/FW) (Figure 5B). Our findings suggest that RAS/ERK pathway activation reduces the differences in miRNA expression profiles between precancerous hepatocytes of the males and females. However, during hepatocarcinogenesis, the differences in miRNA expression profiles between hepatomas of the males and females are enhanced. The 29 overlapping miRNAs between MT/FT and MW/FW are possibly linked to the gender disparity of hepatocytes and hepatomas. Intriguingly, the opposite changes in numbers of up- and down-regulated miRNAs found between MW/FW and MT/FT suggest profound gender disparity of hepatocytes and hepatomas.

### The miRNA Clusters in the Dlk1-Dio3 Genomic Imprinting Region Play Important Roles in Hepatocarcinogenesis

Dlk1-Dio3 GIR is highly conserved in humans and mice, located on chromosome 14q32 and 12qF1, respectively. It contains three protein-coding genes and several non-coding RNA clusters (Figure 5C). The miRNAs encoded in the GIR constitute the largest miRNAs cluster in both human and mouse and play crucial roles in most cancers, through the regulation of multiple pathways (Enfield et al., 2016; Enterina et al., 2017). In the present study, 53.5% of the detected DEMIRs (54 out of 101) between the two sexes were found to be located on Dlk1-Dio3 GIR (Figure 5D and Supplementary Table 9). The variation of DEMIRs in Dlk1-Dio3 GIR is consistent with that of total DEMIRs between sexes (Figures 5A,D), suggesting that the DEMIRs in Dlk1-Dio3 GIR may play crucial roles in gender-disparity of hepatocytes and hepatocarcinogenesis.

Moreover, 54.2% (26 out of 48) DEMIRs in MW/FW were located on Dlk1-Dio3 GIR, and 25 of them were up-regulated in MW (Figures 5D,E and Supplementary Table 9). Intriguingly, in MP/FP, only 5.3% (1 out of 19) DEMIRs, *miR-341-3p*, is located in Dlk1-Dio3 GIR (Figure 5E and Supplementary Table 9). This reflects the reduced gender disparity in miRNA expression in Dlk1-Dio3 GIR under the expression of *Ras* oncogene. However, in MT/FT, 71.6% (53 out of 74) DEMIRs were located in Dlk1-Dio3 GIR (Figure 5E and Supplementary Table 9), and in contrast to MW/FW, 53 miRNAs were down-regulated in MT. During tumorigenesis, the expression of miRNAs located in Dlk1-Dio3 GIR decreased in P and then increase again in T compared to W in males (Figure 5F and Supplementary Table 9). However, in females, the expression of miRNAs in Dlk1-Dio3 GIR tended to increase gradually in P and T compared to W (Figure 5G and Supplementary Table 9). These findings indicate that the overexpression of miRNAs of the Dlk1-Dio3 GIR plays a crucial role in hepatocarcinogenesis in both sexes. The significantly higher levels of these miRNAs in hepatoma in females compared to males imply their important role in the lower susceptibility of hepatocarcinogenesis observed in females.

Although the different expression patterns of miRNAs exist between males and females, the vast majority of DEMIRs located in Dlk1-Dio3 GIR were up-regulated in T comparing to P in both sexes (Figures 5F,G). This finding indicates that the up-regulation of miRNAs located in Dlk1-Dio3 GIR may have similar and important roles in hepatocarcinogenesis in both sexes. In order to understand the functions of these DEMIRs on Dlk1-Dio3 GIR in hepatic tumorigenesis, their target genes were predicted using the TargetScan and miRDB database among the common 530 down-regulated mRNAs in T/P from both sexes (Supplementary Table 10). Target enrichment analysis was performed using the Metascape database. Key metabolic pathways were identified. Among them, pathways related to retinol, steroid, lipid, bile acid (BA), and bile salt were highly enriched (Table 1 and Supplementary Table 10). These results indicate that the miRNA encoded by Dlk1-Dio3 GIR play important roles in hepatocarcinogenesis by regulating multiple metabolic pathways.

**TABLE 1:** Top 20 clusters with their representative enriched terms of target genes of miRNA in DLK1-DIO3 DIR.

**Term**	**Category**	**Description**	**Count^a^**	**%^b^**	**Log10(P)^c^**	**Log10(q)^d^**
R-MMU-211859	Reactome Gene Sets	Biological oxidations	33	12.22	–22.43	–18.17
GO:0032787	GO Biological Processes	Monocarboxylic acid metabolic process	39	14.44	–16.67	–12.72
GO:0017144	GO Biological Processes	Drug metabolic process	42	15.56	–15.33	–11.67
R-MMU-71291	Reactome Gene Sets	Metabolism of amino acids and derivatives	23	8.52	–14.18	–10.62
mmu00830	KEGG Pathway	Retinol metabolism	15	5.56	–12.1	–8.68
GO:0051186	GO Biological Processes	Cofactor metabolic process	30	11.11	–11.43	–8.13
R-MMU-194068	Reactome Gene Sets	Bile acid and bile salt metabolism	11	4.07	–10.24	–7.02
GO:0010817	GO Biological Processes	Regulation of hormone levels	27	10	–8.22	–5.34
mmu00983	KEGG Pathway	Drug metabolism—other enzymes	9	3.33	–7.66	–4.86
GO:0008202	GO Biological Processes	steroid metabolic process	18	6.67	–7.1	–4.41
R-MMU-6788656	Reactome Gene Sets	Histidine, lysine, phenylalanine, tyrosine, proline and tryptophan catabolism	8	2.96	–6.98	–4.31
R-MMU-1614635	Reactome Gene Sets	Sulfur amino acid metabolism	6	2.22	–6.48	–3.87
GO:0016042	GO Biological Processes	Lipid catabolic process	16	5.93	–5.49	–3.03
GO:0006536	GO Biological Processes	Glutamate metabolic process	6	2.22	–5.44	–3
GO:0060749	GO Biological Processes	Mammary gland alveolus development	5	1.85	–5.05	–2.66
R-MMU-192105	Reactome Gene Sets	Synthesis of bile acids and bile salts	6	2.22	–4.99	–2.62
GO:0001505	GO Biological Processes	Regulation of neurotransmitter levels	17	6.3	–4.99	–2.62
GO:0060736	GO Biological Processes	Prostate gland growth	4	1.48	–4.56	–2.24
GO:0050433	GO Biological Processes	Regulation of catecholamine secretion	7	2.59	–4.35	–2.07
GO:1901361	GO Biological Processes	Organic cyclic compound catabolic process	19	7.04	–4.34	–2.07

*^*a*^”Count” is the number of genes in the user-provided lists with membership in the given ontology term.*

*^*b*^”%” is the percentage of all of the user-provided genes that are found in the given ontology term (only input genes with at least one ontology term annotation are included in the calculation).*

*^*c*^”Log10(P)” is the *p*-value in log base 10.*

*^*d*^”Log10(q)” is the multi-test adjusted *p*-value in log base 10.*

## Discussion

Even though miRNAs do not encode for any proteins, they play very important roles in the regulation of gene expression at the post-transcriptional level. The miRNA expression profiles observed in HCC have been extensively studied in the recent years. Multiple profiling studies have revealed that miRNA expression profiles are substantially different between human HCC and healthy livers (Murakami et al., 2006; Jiang et al., 2008). However, the gender disparity of miRNAomics related to hepatic tumorigenesis is largely understudied.

Clinically, due to the limitations of collecting matched specimens from sex-based HCC patients, such as complex etiologies and subclasses, different development stages, difficulties of obtaining normal liver tissues, etc., mechanisms underlying gender-dependent hepatocarcinogenesis are rarely investigated. Therefore, animal models provide valuable opportunities to shed light on clinical investigations for dissecting the underlying mechanisms responsible for this gender disparity. Especially, the matched wild type normal liver tissue and precancerous liver tissue for hepatic tumors provide opportunity for further dynamic change processes analysis during hepatocarcinogenesis. In the present study, we classified the miRNA expression patterns into four categories and several subtypes (Figure 3E), and the following meaningful information is obtained: (1) the DEMIRs classified into (a)-(1) (T > P = W) and (c)-(1) (T < P = W) subtypes indicate that these miRNAs are total absence or weak changes in response to *Ras* expression in precancerous hepatocytes and play predominantly positive and negative roles in hepatic tumors, respectively; (2) the DEMIRs classified into (a)-(2) (T = W > P) and (a)-(3) (T > W > P) subtypes indicate that these miRNAs have a reverse relationship with oncogene expression in precancerous hepatocytes and the expression of these miRNAs is potentially regulated as a part of a tumor defense system; (3) the DEMIRs classified into (c)-(2) (T = W < P) subtype indicates that these miRNAs are positively correlated to *Ras* expression in precancerous hepatocytes and are potentially regulated by the tumor defense system; (4) the DEMIRs classified into (b) and (d) categories indicates that these miRNAs are positive and negative correlation to the *Ras* oncogene in both precancerous hepatocytes and hepatoma cells, respectively. Above novel findings provided valuable clues and insights for further researches.

The miRNAomics data obtained in the present study suggested that, even though the alterations in the miRNA expression profiles during *Ras*-induced hepatic tumorigenesis occurred in a different manner, common changes between males and females were observed as well. Intriguingly, the most profound changes were observed in T when compared to P and W (Figures 3A,B). This was consistent in males and females, and it highlights that miRNAs may play crucial roles in hepatocarcinogenesis. Among all 264 detected DEMIRs, 43 miRNAs (38 up-regulated and 5 down-regulated in T) were found to be related to HCC and were shared between males and females (Figure 3E). Integrating analysis with the RNA sequencing data, the 3-node FFLs were formed and finally filtered out a group of significant components (Figure 4). Among them, *miR-376c-3p*, *miR-664*, *miR-484*, *miR-23a*, *miR-137*, RUNX2, and Ndrg2 had been reported to be closely involved in hepatic tumorigenesis and development (Guo et al., 2013; Liu et al., 2016; Cao et al., 2017; He et al., 2017; Huang B. et al., 2018; Huang H. et al., 2018; Qiu et al., 2018; Wang et al., 2018). It indicates the reliability of the 3-node FFLs analysis. Although the remained genes filtered out in the present study such as *Akr1d, Apoa5, Crp, Cxcl12, Kcnk5, Ngfr, Rnf152, Tmem25, Ptgfrn*, and *Slc6a8* are understudied, the same expression patterns of these genes in mouse and human HCC indicate their important roles in hepatocarcinogenesis and provide a primary reference and potential targets for further investigations.

Deregulation of miRNAs encoded by the Dlk1-Dio3 GIR has been associated with various malignancies, including HCC (Enfield et al., 2016; Enterina et al., 2017). A clinical study reported that the overexpression miRNA found in this cluster was strongly associated with the expression of several HCC stem cell markers, including CD133, CD90, EpCAM, and Nestin. Moreover, high expression levels of these miRNAs correlated with a high level of serum Alpha Fetoprotein (AFP) and worse prognosis in HCC patients (Luk et al., 2011). Additionally, previous studies have demonstrated that miRNAs encoded by the Dlk1-Dio3 GIR, including *miR-127, miR-411, miR-370, miR-134*, and *miR-1188*, are closely associated with the hepatic tumors (Xu et al., 2013; Yin et al., 2013; Cui et al., 2015; Xia et al., 2015; Huan et al., 2016). In agreement with that, most of the DEMIRs identified in the present study were also found to be located encoded by Dlk1-Dio3 GIR (Figure 5). Especially, in our experimental system, we revealed that the miRNA clusters located on Dlk1-Dio3 GIR are closely related to not only hepatic tumorigenesis but also the gender disparity observed in HCC. This new evidence provides an insight in the importance of Dlk1-Dio3 GIR in gender-dependent hepatocarcinogenesis.

The target pathway enrichment analysis revealed that DEMIRs encoded by Dlk1-Dio3 GIR might have a crucial role in driving hepatocarcinogenesis by regulating multiple tumor-associated metabolic pathways (Table 1). Importantly, interference with retinol metabolism may promote hepatocarcinogenesis. Retinol has been reported to exert cytostatic effects in human HCC cells, as well as to promote programmed cell death and cell cycle arrest at the G0/G1 phase (Shimizu et al., 2011). Acyclic retinoid (ACR) has also been reported to have chemopreventive effects in rodent HCC models (Kagawa et al., 2004; Funaki et al., 2017). In line with this, clinical studies have shown that ACR can lead to a significantly lower incident of post-therapeutic recurrence in HCC patients, and as well as to improved outcomes (Muto et al., 1999; Okita et al., 2015). Additionally, the down-regulation of the synthesis of the water-soluble steroids BAs and bile salts may result in the accumulation of cholesterol, which further facilitates the hepatic tumorigenesis and HCC progression. Disruption in BA level balance has been linked to several pathological conditions, including tumorigenesis, suggesting BAs to have potential tumor-promoting roles (Liao et al., 2011). BAs have also been shown to act as strong cues that lead to DNA damage and DNA-damage-induced apoptosis; sustained exposure could, therefore, lead to cells that are less susceptible to apoptosis (Woolbright and Jaeschke, 2012). Moreover, the steroid metabolic process was significantly inhibited. Several steroids, including cholesterol and sex hormones, have been considered to be tumor-related factors. More specifically, cholesterol levels are often higher in HCC cells compared to normal liver cells (Kawata et al., 1990), and the relatively lower serum level of cholesterol observed in HCC patients may be due to cirrhosis, which is observed in 80% of HCC patients (Llovet et al., 2003). Several clinical studies have also provided evidence that cholesterol has a potential contribution to liver malignancies (Montero et al., 2008; Morioka et al., 2016). Androgens are also believed to exert tumor-promoting roles in HCC, while estrogen is believed to have a protective role. Therefore, sex hormones not only play crucial roles in regulating the differences in sex characteristics and biological processes between males and females but are also believed to contribute to gender-dependent hepatocarcinogenesis (Yeh and Chen, 2010). In summary, the target pathway enrichment analysis shows that DEMIRs on Dlk1-Dio3 GIR may affect hepatocarcinogenesis by regulating multiple tumor-associated metabolic pathways.

The *Hras12V*-transgenic hepatic cancer mouse model has been previously described in detail (Wang et al., 2005). Briefly, *Hras12V*-transgenic mice express the liver-specific *Hras12V* oncogene, resulting in liver cancer development at the definite stage and with considerable gender disparity. Namely, compared to *Ras*-Tg males, the hepatic tumorigenesis in females is characterized by late-onset, low incidence, slow progression, and small tumor size. In detail, male *Ras*-Tg mice develop HCC relatively stage, usually between 8 and 9 months of age); the incidence of HCC in these mice is extremely high (closed to 100%). HCC tends to progress rapidly, and the mice eventually die of hepatic tumors at 12–14 months of age. In contrast, female *Ras*-Tg mice develop hepatic tumors at a much lower rate (∼30%), and this happens at a much later stage (15-month-old) compared to male mice. Therefore, although age-related factors may be involved, to investigate the miRNAomics involved in the *Ras-*driven gender-dependent HCC, male and female mice aged 9- and 15-months were must be used, respectively. Our investigation had indicated that differences in molecular responses to deregulated RAS oncoprotein between *Ras*-Tg females and males determine the onset of HCC development (Wang et al., 2005). Further, by exploring the advanced omics-based molecular techniques, our previously published global proteomics and metabonomics data had revealed gender-dependent routes for hepatocarcinogenesis (Fan et al., 2017; Rong et al., 2017). In the present study, the gender disparities were also reflected in the miRNAomics data. As the humans and rodents were supposed to share the common routes for gender dependent HCC, the *Ras*-Tg combined with its related global and systemic omics-data will provide a valuable model for elucidating the underlying mechanisms contributing to the gender disparity of hepatocarcinogenesis.

In conclusion, to our best knowledge, this is the first study to present global miRNAomics data related to gender disparity in *Ras*-induced HCC. The shared and gender-dependent biological and molecular changes that occur during hepatic tumorigenesis offer valuable clues in elucidating the mechanisms that contribute to male-biased *Ras*-induced hepatocarcinogenesis. The differentially expressed miRNAs identified in the hepatic tumors will add important biological information to tumor-related bioinformatics databases. Particularly, the overexpression of miRNA clusters on Dlk1-Dio3 GIR was identified as a promoting factor in hepatocarcinogenesis by regulating multiple key cellular pathways.

## Materials and Methods

### Animals Handing and Tissue Sampling

Procedures involving animal maintenance and handling, as well as tissue sampling were approved by the Animal Care and Use Committee of Dalian Medical University. Non-transgenic C57BL/6J mice (Non-Tg, purchased from Institute of Genome Engineered Animal Models for Human Disease in Dalian Medical University) and *Hras12V*-transgenic mice (*Ras*-Tg, a kind gift from Dr. Dae-Yeul Yu at Korea Research Institute of Bioscience and Biotechnology) were used for this study. Both strains were bred and housed in pathogen-free animal facility of the Laboratory Animal Center of Dalian Medical University. All animals were fed a standard normal diet (Liaoning Changsheng Biotechnology Co., Ltd.) *ad libitum* with free access to water and housed under controlled temperature (around 23°C) and relative humidity (40–60%) conditions with a 12–12 h light-dark cycle. To assess the male bias of hepatic tumorigenesis of *Ras*-Tg, 9-month-old male and 15-month-old female *Ras*-Tg bearing hepatic tumors of the same stage were harvested. Healthy liver tissues from non-Tg males and females were also sampled as control. After the mice were culled and subjected to euthanasia by cervical dislocation, normal liver tissue samples from male and female non-Tg (W) and hepatic tumors (T) and matched adjacent precancerous tissue samples (P) of *Ras*-Tg were collected. Tissue samples were flash-frozen in liquid nitrogen. Tissue samples were also fixed in 10% formalin and subsequently used for histopathological examination.

The pathological diagnosis confirmed tissues were selected for the following experiments. The precancerous and HCC tissues of *Ras*-Tg were used in the present study. The precancerous tissues are the morphological normal liver tissues between hepatic tumors, in which the hepatocarcinogenesis is induced by hepatocyte-specifically expressed *Hras12V* oncogene. Microscopic findings of the precancerous liver tissues showed largely the same as normal liver tissues, but with scattered distribution of histopathological changes including fat vacuoles, necrosis, edema, and altered hepatic foci surrounding the central vein of hepatic lobule. The pathological characteristics of HCC were large in size, typically more than 5 mm in diameter; showed a trabecular arrangement of tumor cells; and contained highly anaplastic cells with evidence of necrosis.

### Experimental Design

Seven representative individuals were selected from each group, and small RNA samples were prepared individually. Mixing samples of experimental animal tissues of the same group is one of the most frequently used methods of quantitative sequencing, especially for inbred strains, which are nearly identical to each other in terms of genomic background. In addition, in the case of *Ras*-Tg, the hepatic tumors were protruded from the peri-tumor tissues and had a clear boundary between them, allowing for precise sampling yet a small chance to mix the hepatic tumor tissues with the peri-tumor tissues does exist. Therefore, seven individual samples of the same group were equivalently mixed to generate one combined sample. This design ensures sample coverage and reliable NGS results, which reflect the shared changes during hepatic tumorigenesis with a male bias. NGS approaches were performed to quantify the miRNAomics for MW, MP, MT, FW, FP, and FT. The workflow is depicted in Supplementary Figure 1.

### Quantitative miRNAomics Analysis by NGS

MiRNA expression profiles were assessed using NGS analysis. miRNAs were isolated from W, P, and T tissues (seven individuals for each group); The RNA extraction was conducted using the mirVana^TM^ miRNA Isolation Kit (AM1561, Austin, TX, United States) according to the manufacturer’s protocols. The total RNA quality and quantity were verified by electrophoresis using an Agilent 2100 Bioanalyzer (Agilent Technologies, Santa Clara, CA, United States). Due to the fact that the genetic background of the mice used, the common hepatocarcinogenesis etiology (*Ras*-driven), and the high similarity in the pathological characteristics of the samples from each group, the RNA samples from the same group were mixed at equivalent proportions to generate six composite samples, i.e., W of male and female non-Tg (MW and FW, respectively), P of male and female *Ras*-Tg (MP and FP, respectively), and T of male and female *Ras*-Tg (MT and FT, respectively), to find out common variations against interindividual differences in the same group. MiRNA libraries were constructed with the TruSeq Small RNA Sample Prep Kit (Illumina, San Diego, CA, United States). The Illumina HiSeq 2000 (Illumina, San Diego, CA, United States) instrument was employed to sequence the miRNA library. Contaminating and low-quality reads were removed using the Fastx software (version: 0.0.13).^1^

### Identification of Differentially Expressed miRNAs

The RNA-seq data were used to identify the DEMIRs. Firstly, clean reads of 18--44 nt were mapped onto the reference genome (GRCm38)^2^ using Bowtie (Langmead et al., 2009). MiRNAs and other small RNAs were identified according to the location information of known miRNAs in miRBase, as described previously (Kozomara and Griffiths-Jones, 2014), and the location information of other small RNAs in the reference genome. In order to make the miRNA expression levels between different miRNAs and between different samples comparable, the reads of each miRNA were normalized using the trimmed mean of M values (TMM) method (Robinson and Oshlack, 2010) and then converted into TPM (Transcripts per million; the formula used is: reads number on a miRNA × 10^6^/total reads number) for the standardization (Mortazavi et al., 2008). Fold-change was calculated according to TPM, and PCA was performed. EdgeR (Robinson et al., 2010) was used to identify DEMIRs in paired comparisons between W, P, and T, using the mapped reads. Filtering was performed using q-value ≤ 0.05 and fold-change ≥ 2 as threshold.

### Validation of DEMIRs by RT-qPCR

To confirm the validity and reproducibility of our miRNAomics analysis findings, six of the identified differentially expressed miRNAs in both sexes were randomly selected and further evaluated by RT-qPCR analysis in samples from another set of males and females. The cDNA was synthesized using the Mir-X miRNA First-Strand Synthesis Kit (TaKaRa, Dalian, China) in accordance with the manufacturer’s instructions. RT-qPCR reactions were conducted with a StepOnePlus^TM^ Real-Time PCR System (Thermo Fisher Scientific, CA, United States) using SYBR Green qPCR Master Mix (TaKaRa, Dalian, China). Mouse small nucleolar RNA, C/D box 68 (*Snord68*) was used as a reference gene for normalization (Mu et al., 2013). The amount of miRNA relative to the internal control *Snord68* was calculated using the ΔΔCt method (2^–(*Ct* miRNA^ ^– C*t* Snord68)^). The primers for the miRNAs were synthesized by Dalian Saituo biotechnology company (Dalian, China). The primer sequences are shown in Supplementary Table 11.

### Identification of Common Regulatory Networks Related to Hepatocarcinogenesis

Differential mRNA expression analysis by NGS has been reported in our previously published study (Huan et al., 2016). Considering that mRNAs are negatively regulated by miRNAs, the 3-node FFL co-regulatory network was built by exploring miRNA and mRNA-omics. Firstly, the miRNA-gene pairs were extracted using the TargetScan (release 7.1) (Agarwal et al., 2015) and miRDB (Wong and Wang, 2015) database. The miRNA-mRNA pairs with a total context score <–0.3 in TargetScan and target score >50 in miRDB were extracted. The two sets of miRNA-gene pairs were merged, and the mRNAs present in both databases were considered to be miRNA targets. Transcription factors (TFs) were retrieved from differently expressed mRNAs using the TRANSFAC Professional Database (release 2018.2) (Dubchak et al., 2013). To assess the regulation of miRNA and mRNAs by TFs, the miRNA promoter region sequences (–2,000 around TSS) and the promoter region sequences (–2,000/+500 around TSS) of genes involved in HCC were obtained from the UCSC database. These sequences were subsequently investigated for TF-binding sites, using the TRANSFAC Professional Database. Pre-calculated cut-offs were used to eliminate false-positive (minFP) matches and construct a high-quality matrix. We also limited the search to a core score of 1.00, matrix score > 0.95, and TFs that only belong to the mouse genome. Finally, four types of regulatory relationships among miRNAs, TFs, and genes were predicted.

The 3-node feed-forward loop (3-node FFL) is extremely common among transcriptional networks, and it can be broadly divided into three categories based on their inside regulations: miRNA-FFL, TF-FFL, and composite-FFL. In TF-FFL, the TF regulates the miRNA and the gene, while the miRNA represses the target gene. In miRNA-FFL, the miRNA represses both TF and gene expression while the TF regulates target gene expression. In composite-FFL, the TF regulates the miRNA and target gene while the miRNA represses the TF and the gene. The three types of FFLs are exclusive to each other. Therefore, three types of FFLs (miRNA-FFL, TF-FFL, and composite FFL) were extracted from the four categories of regulation relationships, and then the sub-networks (SNWs) were visualized using Cytoscape (version 3.6.1).

### Identification of Significant Components

In the three SNWs, TF-SNW is the most redundant. To focus on the most significant components, the top 25% degree nodes of gene, miRNA and TF were determined as the member of secondary TF-SNW by using the hub definition method (Yu et al., 2004). All nodes in the three SNWs (secondary TF-SNW, miRNA-SNW, composite-SNW) were filtered based on the GEPIA (Tang et al., 2017) and PubMed database. The nodes have the same expression trend in human GEPIA database or their roles in hepatocarcinogenesis elucidated were considered to be significant components.

### Pathway Enrichment Analysis

The target pathway enrichment analysis was performed by Metascape database (Zhou et al., 2019).

## Data Availability Statement

The datasets presented in this study can be found in online repositories. The names of the repository/repositories and accession number(s) can be found below: [NCBI SRA and BioProject PRJNA670622].

## Ethics Statement

The animal study was reviewed and approved by the Animal Care and Use Committee of Dalian Medical University.

## Author Contributions

AW and JW designed the experiments, provided research funds, and corrected the manuscript. JZ, HL, JD, NZ, YL, XL, JC, and AW performed the experiments and analyzed the data. JZ, HL, and AW co-wrote the manuscript. All authors read and approved the final manuscript.

## Conflict of Interest

The authors declare that the research was conducted in the absence of any commercial or financial relationships that could be construed as a potential conflict of interest.

## Footnotes

1 http://hannonlab.cshl.edu/fastx_toolkit/index.html

2 ftp://ftp.ensembl.org/pub/release-83/fasta/mus_musculus/dna/Mus_musculus.GRCm38.dna.primary_assembly.fa.gz

## Supplementary Material

Supplementary Figure 1 Experimental flowchart. The miRNA and mRNA expression profiles of tissue samples from male and female mice were determined by next-generation sequencing. Following vertical and horizontal comparison, expression profiling, 3-node-FFL, DLK1-DIO3 GIR, and pathway enrichment analysis were performed. MW and FW, wild-type liver tissues of males and females, respectively; MP and FP, peri-tumor tissues of males and females, respectively; MT and FT, hepatic tumor tissues of males and females, respectively; “/”, comparison between former and latter; DEMIRs, differentially expressed miRNAs; DEMIRs, differentially expressed mRNAs; GIR, genomic imprinting region; FFL, feed-forward loop.

Supplementary Figure 2 Saturation analysis for detected miRNAs mapped by clean reads.

Supplementary Figure 3 The expression pattern analysis of total DEMIRs for hepatocarcinogenesis. The description is same as Figure 4E. Specially, the white column in expression models indicates that the miRNA levels in this tissue have no significant difference compared with other tissues.

Supplementary Table 1 Raw and clean reads obtained by NGS.

Supplementary Table 2 DEMIRs in males (191) and females (204).

Supplementary Table 3 Common and unique DEMIRs in different expression patterns.

Supplementary Table 4 Up-regulated and down-regulated mRNAs selected for the 3-node FFL co-regulatory network analysis.

Supplementary Table 5 Relationships between HCC-related genes, miRNAs, and TFs.

Supplementary Table 6 Relationships between TFs, miRNAs, and genes.

Supplementary Table 7 Summary of 3-node FFL based on CRC-related prediction data.

Supplementary Table 8 HCC-related genes in miRNA-FFLs, TF-FFLs, and composite-FFLs.

Supplementary Table 9 DEMIRs in the Dlk1-Dio3 DIR.

Supplementary Table 10 Metascape analysis for down-regulated mRNAs corresponding to up-regulated miRNAs in the Dlk1-Dio3 DIR of both males and females.

Supplementary Table 11 The miRNA specific 5’-primer sequences of selected DEMIRs and internal control.

## Abbreviations

DEMIRs: differentially expressed miRNAs
F: females
FW: normal liver tissues of non-transgenic 15 months old female mice
FP: normal peri-tumor precancerous tissues of transgenic 15 months old female mice
FT: hepatic tumor tissues of transgenic 15 months old female mice
GIR: genomic imprinting region
HCC: hepatocellular carcinoma
M: male
miRNA: microRNA
MP: normal peri-tumor precancerous tissues of transgenic 9 months old male mice
MW: normal liver tissues of non-transgenic 9 months old male mice
MT: hepatic tumor tissues of transgenic 9 months old male mice
NGS: next generation sequencing
non-Tg: C57BL/6J wild type non-transgenic mice
P: peri-tumor precancerous tissue of *Ras*-Tg
PCA: principal component analysis
P/W: P versus W
3-node FFL: 3-node feed-forward loop
SNWs: sub-networks
T: hepatic tumor of *Ras*-Tg
TPM: transcripts per million
T/P: T versus P
T/W: T versus W
*Ras*-Tg: *Hras12V* transgenic mice
W: normal liver tissues of non-Tg.
