# Supplementary figures and images for: Omics-Based Identification of Shared and Gender Disparity Routes in Hras12V-Induced Hepatocarcinogenesis: An Important Role for Dlk1-Dio3 Genomic Imprinting Region
Source: Front Genet. 2021 May 31;12:620594. doi: 10.3389/fgene.2021.620594 (PMC8202007; doi:10.3389/fgene.2021.620594)

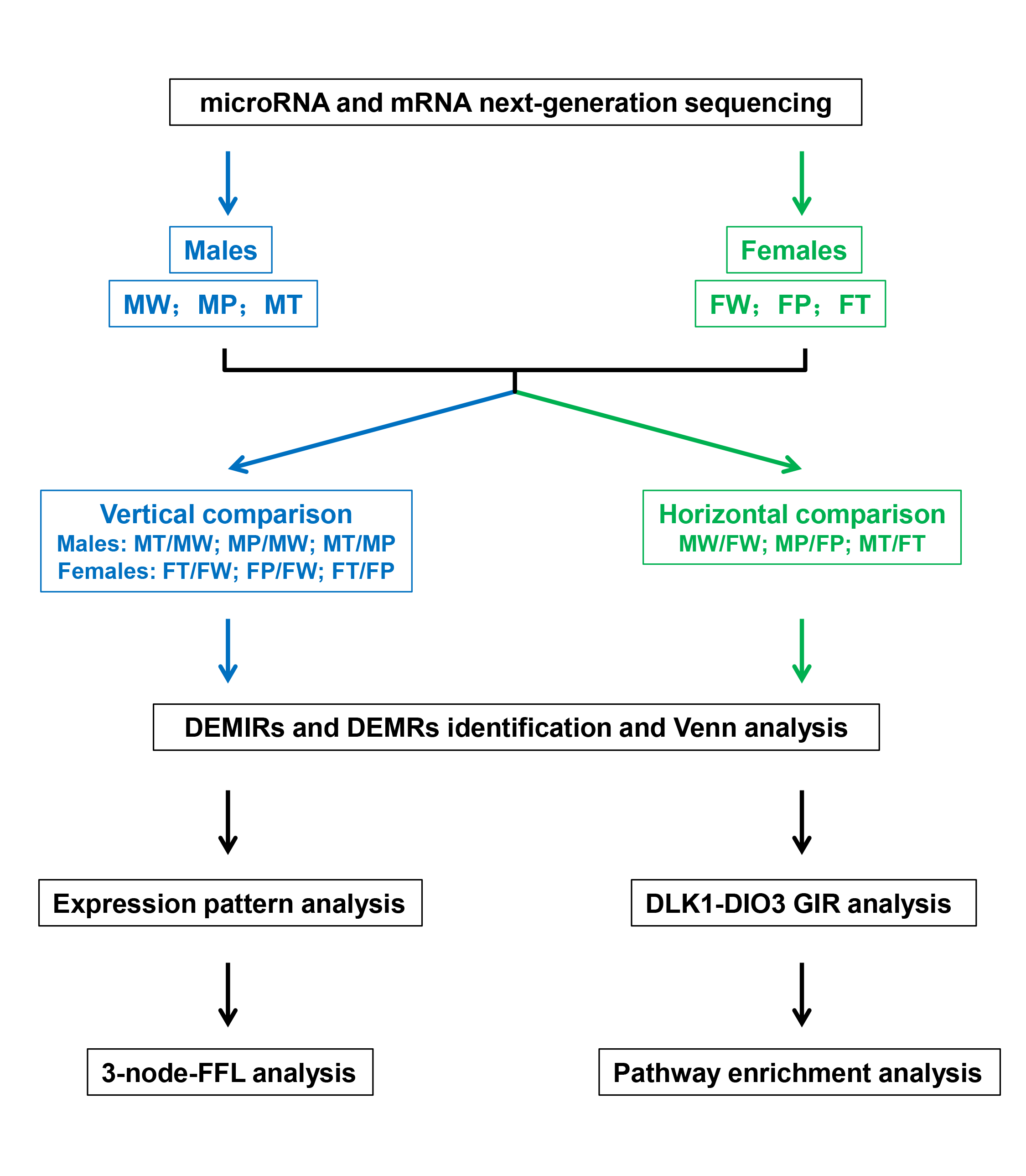

Supplement: Supplementary Figure 1 — Experimental flowchart. The miRNA and mRNA expression profiles of tissue samples from male and female mice were determined by next-generation sequencing. Following vertical and horizontal comparison, expression profiling, 3-node-FFL, DLK1-DIO3 GIR, and pathway enrichment analysis were performed. MW and FW, wild-type liver tissues of males and females, respectively; MP and FP, peri-tumor tissues of males and females, respectively; MT and FT, hepatic tumor tissues of males and females, respectively; “/”, comparison between former and latter; DEMIRs, differentially expressed miRNAs; DEMIRs, differentially expressed mRNAs; GIR, genomic imprinting region; FFL, feed-forward loop. [file Data_Sheet_1.ZIP › Supplementary files/Figure S1.tif]

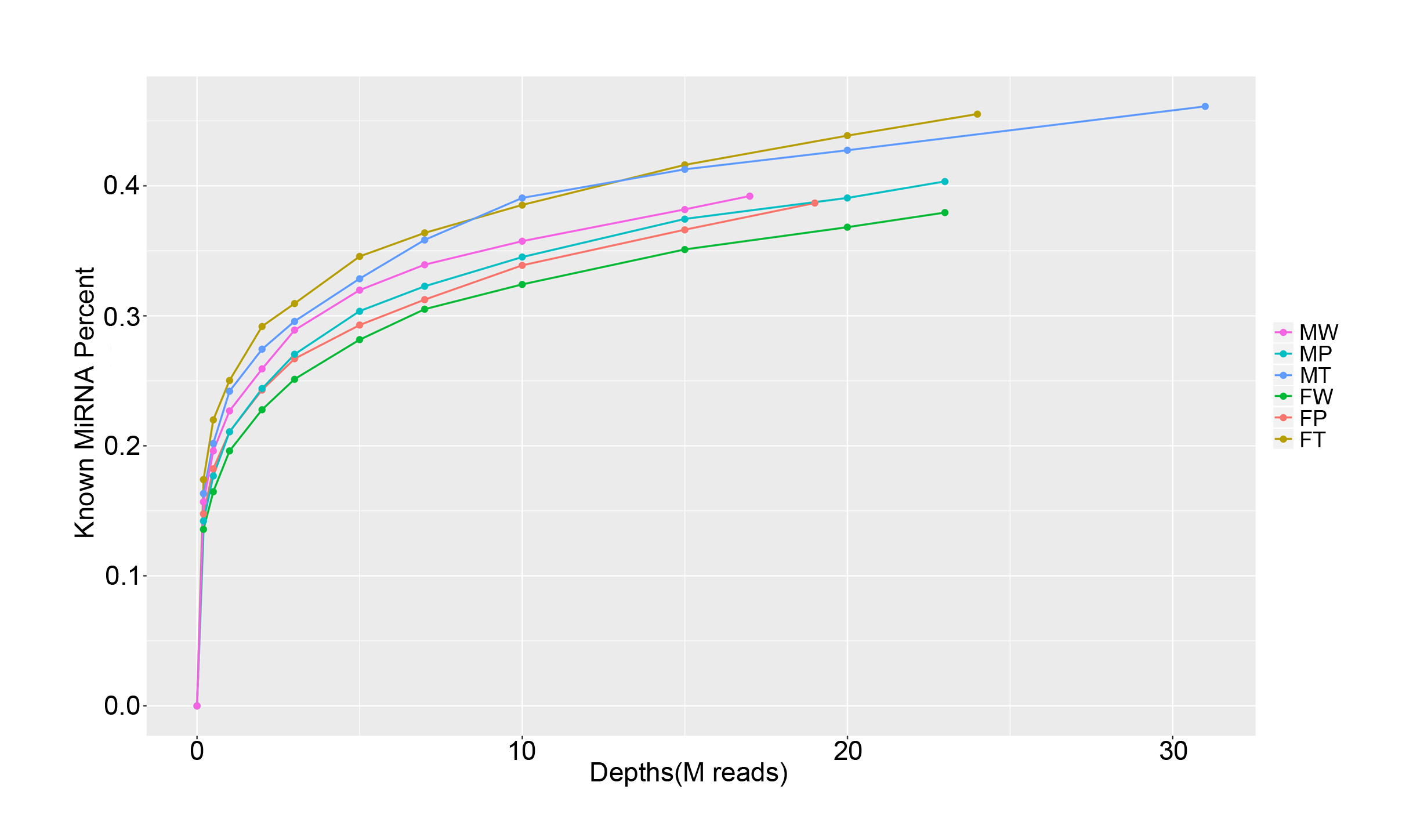

Supplement: Supplementary Figure 1 — Experimental flowchart. The miRNA and mRNA expression profiles of tissue samples from male and female mice were determined by next-generation sequencing. Following vertical and horizontal comparison, expression profiling, 3-node-FFL, DLK1-DIO3 GIR, and pathway enrichment analysis were performed. MW and FW, wild-type liver tissues of males and females, respectively; MP and FP, peri-tumor tissues of males and females, respectively; MT and FT, hepatic tumor tissues of males and females, respectively; “/”, comparison between former and latter; DEMIRs, differentially expressed miRNAs; DEMIRs, differentially expressed mRNAs; GIR, genomic imprinting region; FFL, feed-forward loop. [file Data_Sheet_1.ZIP › Supplementary files/Figure S2.tif]

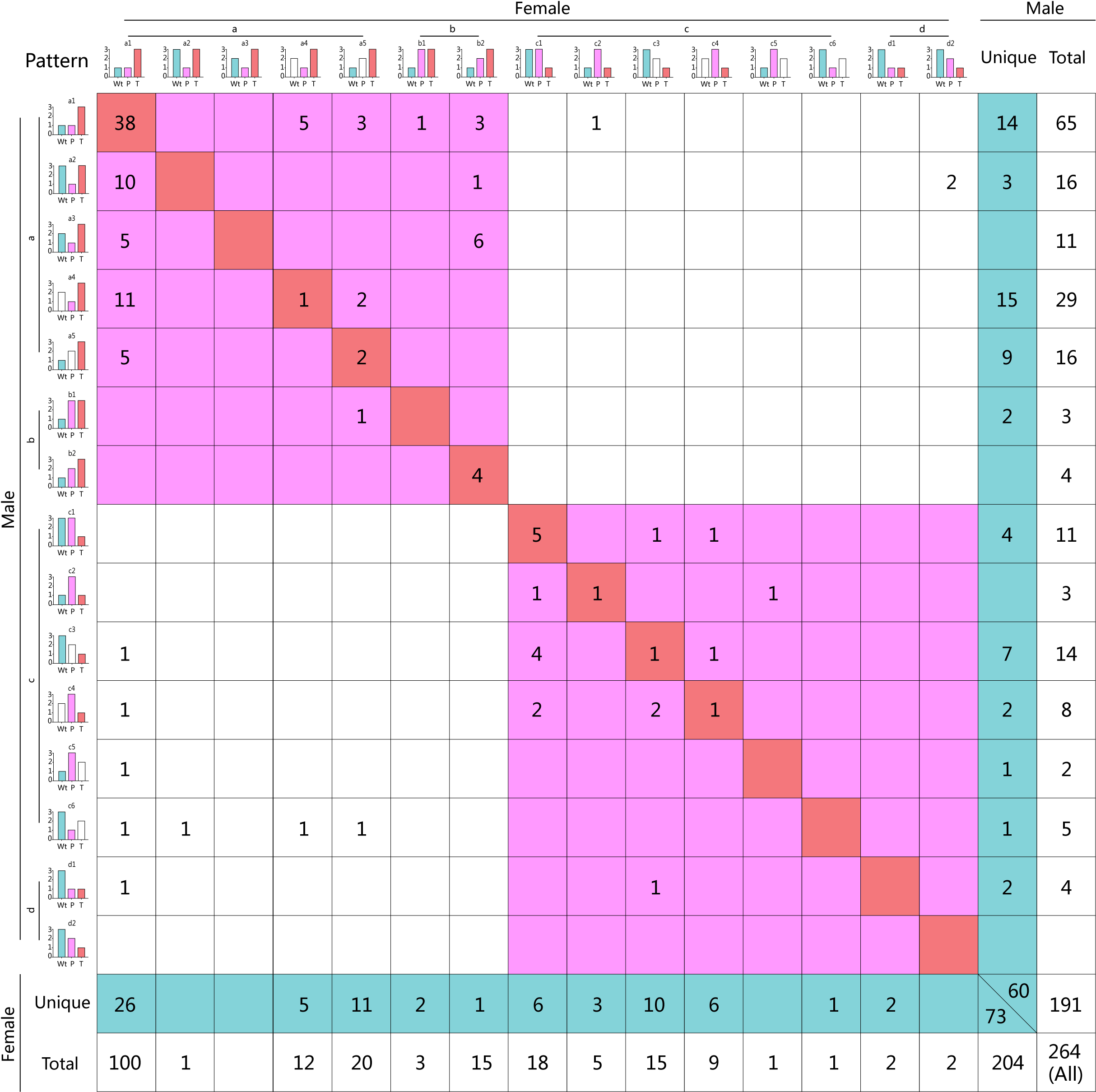

Supplement: Supplementary Figure 1 — Experimental flowchart. The miRNA and mRNA expression profiles of tissue samples from male and female mice were determined by next-generation sequencing. Following vertical and horizontal comparison, expression profiling, 3-node-FFL, DLK1-DIO3 GIR, and pathway enrichment analysis were performed. MW and FW, wild-type liver tissues of males and females, respectively; MP and FP, peri-tumor tissues of males and females, respectively; MT and FT, hepatic tumor tissues of males and females, respectively; “/”, comparison between former and latter; DEMIRs, differentially expressed miRNAs; DEMIRs, differentially expressed mRNAs; GIR, genomic imprinting region; FFL, feed-forward loop. [file Data_Sheet_1.ZIP › Supplementary files/Figure S3.tif]
